# Sentiment Analysis of Cochlear Implants in the r/Cochlear Implants Subreddit

**DOI:** 10.1002/oto2.70165

**Published:** 2025-09-19

**Authors:** Rose Dimitroyannis, Ethan Oliver, Ringo Leung, Rachel Nordgren, Terence E. Imbery

**Affiliations:** ^1^ Pritzker School of Medicine University of Chicago Chicago Illinois USA; ^2^ Chicago Medical School Rosalind Franklin University of Medicine and Science North Chicago Illinois USA; ^3^ Department of Public Health Sciences University of Chicago Chicago Illinois USA; ^4^ Department of Surgery, Section of Otolaryngology–Head and Neck Surgery University of Chicago Chicago Illinois USA

**Keywords:** Cochlear Implant, internet, sentiment analysis

## Abstract

**Objective:**

Cochlear implants (CI) are surgical devices used for rehabilitation of sensorineural hearing loss. More individuals are receiving CIs as technology and surgical techniques improve and candidacy guidelines expand. Despite growing public awareness, CI utilization remains low. Understanding sentiment regarding these devices is important. Reddit is a text‐based website wherein posters interact on specialized forums, “subreddits.” R/Cochlearimplants allows for unique CI sentiment analysis.

**Study Design:**

A search was done from October 2024 to November 2024 on r/Cochlearimplants.

**Setting:**

Internet.

**Methods:**

Posts were sorted by highest engagement. Metadata regarding date, comments, and upvotes were collected. Sentiment was analyzed using TextBlob and VADER, Python library Natural Language Processing tools.

**Results:**

Four hundred and twenty unique posters made 1068 total entries. Entries spanned 2019 to 2024, the majority in 2024 (51%, n = 543). VADER found the majority of entries positive (n = 562, 52.9%) while TextBlob found the majority neutral (n = 928, 87%). Sentiment distribution over time was significantly different (*P* < .001), with more negative sentiment in 2024 than 2019 to 2023. Negative VADER entries had significantly higher word counts (*P* < .001). Positive VADER entries had higher upvotes (*P* < .001).

**Conclusion:**

Sentiment regarding CI remains more nuanced than can be gleaned from this analysis, including cultural and ethical issues. This study demonstrates that sentiment on r/Cochlearimplants is generally neutral or positive, trending relatively more negative over time. This could suggest negativity towards CIs is growing with increased utilization. Awareness of online sentiment may help providers understand patient perspectives and dispel misinformation.

Cochlear implants (CI) are surgically implanted electrical devices that provide hearing rehabilitation to adults and children with significant sensorineural hearing loss.^1^ As of July 2022, over 1 million registered CIs have been implanted worldwide.^2^ With the prevalence of hearing loss expected to rise over time, and lack of an alternative treatment for profound sensorineural hearing loss, the number of CI candidates is anticipated to increase.^3^, ^4^ Additionally, as CI surgical techniques are optimized, candidacy is expanded, telemedicine reaches more patients, and CI technology improves, more individuals than ever are predicted to utilize CIs.^5^, ^6^ CI intervention has been linked with improved quality of life across multiple domains and positively impacted patients’ lives postoperatively.^7^

Despite the benefits CIs provide, they still have limited overall utilization.^8^ The reasons for this are multifactorial and include concerns about the loss of residual hearing, long‐term outcomes, accessibility, and so forth.^9^ Furthermore, some view deafness as a cultural identity rather than a condition to be treated, leading to ethical or personal reservations regarding CI. Improving our understanding of patients' and family members' experiences and perceptions of CIs throughout all stages of their CI journey may improve outcomes and address misinformation. Prior studies have highlighted the usefulness of social media in understanding patient experiences. For example, one study utilized Instagram and the hashtag #headandneckcancer to gain insight into the daily experiences of patients, which provided information that is crucial to providing patient‐centered, comprehensive care.^10^ To date, there is limited research using social media platforms to understand sentiments and perceptions around CIs.^11^

Reddit is a popular social media platform that allows users to create entries and interact with each other within specialized forums known as “subreddits” on almost any topic. Reddit allows individuals with common interests or shared experiences to create a community without physical or geographic limitations.^12^ R/Cochlearimplants is the dedicated subreddit for users to create and discuss entries on topics related to CIs. Individuals may seek information, opinions, advice, troubleshooting, and support related to CIs and hearing loss. Due to the anonymity that Reddit provides, individuals may feel more comfortable giving their unfiltered opinions regarding CIs or disclosing medical information compared to other social media platforms.^13^, ^14^ Given that the CI subreddit provides a platform for many individuals across different backgrounds to discuss CIs, it may provide a valuable source of information for clinicians and researchers. Therefore, this study aims to analyze the sentiments and contents of entries within the r/Cochlearimplants subreddit to understand patient and family member perspectives and feelings toward CI intervention.

## Methods

### Data Collection

Subreddits are text forums pertaining to a specific topic. Within the forum, users can create entries, both posts and comments on other users' posts. Users can also engage with entries by voting on them, “upvoting” those that are found to be useful and “downvoting” those that are not. The cumulative score on an entry is shown to users, taking into account up and downvotes. Collection of entries from the r/CI subreddit was performed from 10/1/2024 to 11/1/2024, but included entries dating back to the site's inception in 2015. Posts were sorted and filtered by “top” and “all time” to view posts with the largest amount of cumulative upvotes throughout the site's archive. The r/Cochlearimplants subreddit site was first used on March 1, 2015. Posts with 30 or greater upvotes and their adjoining comments were collected to guarantee the incorporation of impactful discussions. Metadata for each entry was collected, including uniform resource locator (URL), date posted, word count, number of upvotes, and number of comments.

### Statistical Analysis

Descriptive statistics were conducted using the Kruskal‐Wallis test for continuous variables and the chi‐squared test for categorical variables. Statistical analyses were performed using Excel 16, R (version 4.3.2; Foundation for Statistical Computing), and Python (version 3.11.5).^15^, ^16^, ^17^, ^18^, ^19^ A significance threshold of *α* = 0.001 was used to account for the presence of multiple outcomes. Sentiment within posts was analyzed using TextBlob and VADER (Valence Aware Dictionary and Sentiment Reasoner), two Python library tools using Natural Language Processing to assess the polarity of text.^20^, ^21^ While both tools are equipped for sentiment analysis, VADER was created to account for social media vernacular; therefore, VADER sentiment scores are affected by capital letters, repeated words, or emojis.^22^ Sentiment scores range from −100 to +100, with −100 representing negative, 0 representing neutral, and +100 representing positive.

### Ethical Considerations

This research was found to be exempt by the University of Chicago Institutional Review Board (IRB), IRB24‐1632.

## Results

A total of 1068 entries from the r/Cochlearimplants subreddit were analyzed, comprising 53 original posts (5%) and 1015 comments. These entries were contributed by 424 unique posters. The dataset spans from 2019 to 2024, with the majority of entries (51%, n = 543) posted in 2024. The mean number of words per entry was 52, and posts received an average of 19.4 comments (Table 1).

**Table 1:** Metadata Regarding Subreddit and Sentiment Analysis Using VADER and TextBlob

Number of entries (posts + comments)	1068
Number of posts	53
Years posted	2019‐2024
Number of unique posters	424
Mean number of words per entry	52 (SE = 2.9)
Mean number of comments per post	19.4 (SE = 2.6)
VADER positive:neutral:negative ratio	53:41:6
TextBlob positive:neutral:negative ratio	11:87:2

**Figure 1:**
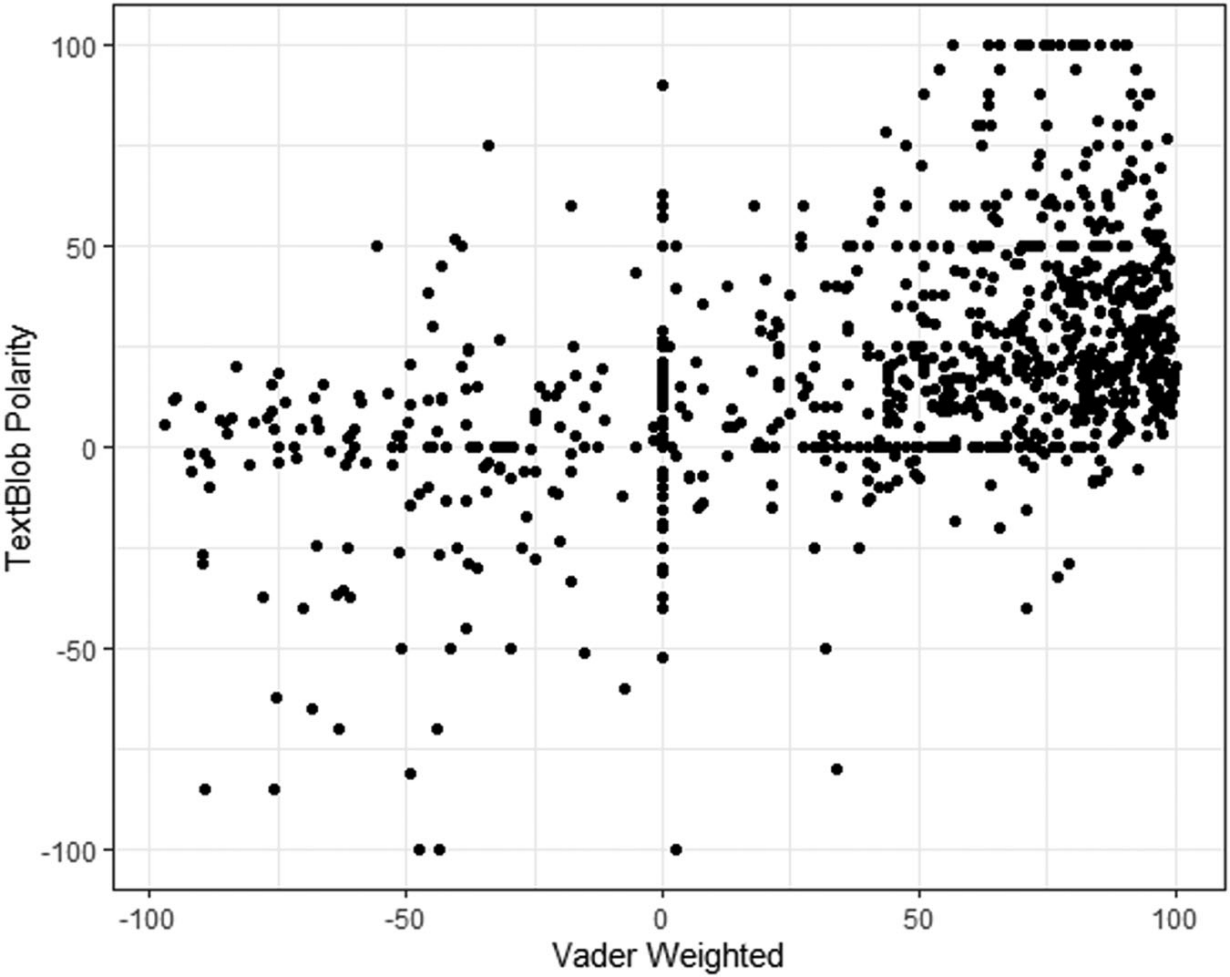
VADER versus TextBlob sentiment scores. −100 represents negative sentiment, +100 represents positive sentiment, 0 represents neutral sentiment.

**Figure 2:**
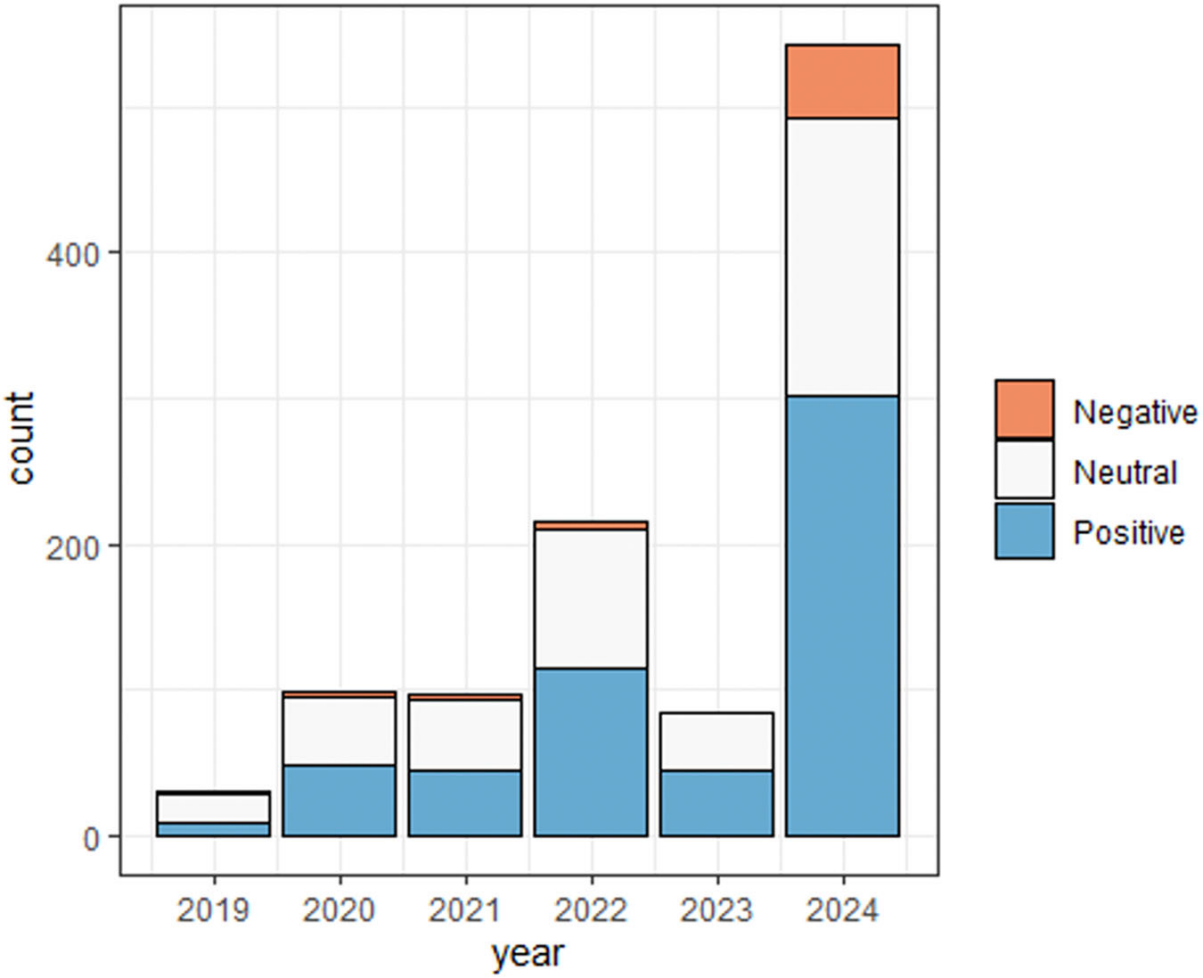
Sentiment over time on r/Cochlearimplants. Sentiment using VADER.

Sentiment analysis using VADER and TextBlob revealed differing classifications. VADER categorized 53% of entries as positive, 41% as neutral, and 6% as negative. In contrast, TextBlob identified 87% of entries as neutral, 11% as positive, and only 2% as negative. (Figure 1) A significant shift in sentiment distribution was observed over time (*P* < .001), with 75% of negative sentiment posts occurring in 2024 compared to previous years (2019‐2023) (Figure 2). Additionally, negatively classified entries had significantly more words than neutral or positive ones (*P* < .001), with a median of 66 words compared to 16 and 33 words, respectively (Table 2).

**Table 2:** Significance Testing for Sentiment Analysis, Using VADER Categories

	Vader negative (N = 65)	Vader neutral (N = 441)	Vader positive (N = 562)	Total (N = 1068)	*P* value
Year					<.001
2019	1 (2%)	20 (5%)	9 (2%)	30 (3%)	
2020	4 (6%)	47 (11%)	48 (9%)	99 (9%)	
2021	3 (5%)	49 (11%)	44 (8%)	96 (9%)	
2022	5 (8%)	96 (22%)	114 (20%)	215 (20%)	
2023	1 (2%)	39 (9%)	45 (8%)	85 (8%)	
2024	51 (78%)	190 (43%)	302 (54%)	543 (51%)	
Number of words					<.001
Median (Q1, Q3)	66.0 (24.0, 144.0)	16.0 (8.0, 31.0)	33.0 (16.0, 69.8)	25.0 (11.0, 55.0)	
Mean (SE)	98.1 (12.3)	25.2 (1.3)	67.7 (5.0)	52.0 (2.9)	
Type of entry (Post)	2 (3%)	17 (4%)	34 (6%)	53 (5%)	.218
Number of comments					.518
N‐Miss	63	424	528	1015	
Median (Q1, Q3)	52.0 (31.0, 73.0)	12.0 (8.0, 16.0)	13.5 (10.0, 21.0)	13.0 (9.0, 21.0)	
Mean (SE)	52.0 (42.0)	18.6 (4.9)	17.9 (2.3)	19.4 (2.6)	
Number of comments from OP					.451
N‐Miss	63	425	534	1022	
Median (Q1, Q3)	6.0 (3.0, 9.0)	1.5 (0.0, 3.8)	3.0 (1.0, 6.0)	2.0 (1.0, 6.0)	
Mean (SE)	6.0 (6.0)	3.9 (1.6)	4.0 (0.7)	4.1 (0.7)	
Number of upvotes (cumulative)					<.001
Median (Q1, Q3)	2.0 (1.0, 4.0)	2.0 (1.0, 3.0)	2.0 (1.0, 4.0)	2.0 (1.0, 4.0)	
Mean (SE)	4.2 (0.8)	4.3 (0.5)	5.3 (0.5)	4.8 (0.3)	
Picture	1 (2%)	16 (4%)	17 (3%)	34 (3%)	.638
Weighted Vader					<.001
Median (Q1, Q3)	−70.4 (−83.0, −61.2)	0.0 (0.0, 33.8)	80.7 (67.0, 90.5)	54.6 (0.0, 81.7)	
Mean (SE)	−71.6 (1.6)	7.2 (1.3)	78.8 (0.6)	40.0 (1.5)	
TextBlob sentiment					<.001
Median (Q1, Q3)	0.0 (−10.0, 6.8)	0.0 (0.0, 16.7)	24.7 (12.5, 45.0)	14.3 (0.0, 31.7)	
Mean (SE)	−7.5 (3.2)	6.4 (1.1)	31.4 (1.1)	18.7 (0.9)	
TextBlob Category					<.001
Negative	6 (9%)	12 (3%)	0 (0%)	18 (2%)	
Neutral	59 (91%)	410 (93%)	459 (82%)	928 (87%)	
Positive	0 (0%)	19 (4%)	103 (18%)	122 (11%)	

Among the 424 unique posters, Vader sentiment distribution was 3.8% (n = 16) negative, 44.3% (n = 188) neutral, and 51.9% (n = 220) positive. Posts with negative sentiment were more likely to include detailed narratives or concerns regarding CI, whereas positively classified posts frequently highlighted successful implantation experiences and improvements in quality of life. Notably, positive posts received significantly more upvotes than neutral or negative entries (*P* < .001), indicating stronger engagement with optimistic content.

Further breakdown by posting behavior through Vader revealed that users with negative sentiment were less likely to have engaged in long‐term posting (>1 year) (0%, *P* = .002). The majority of users with negative sentiment (75%) started posting in 2024, while positive sentiment was more evenly distributed across years. The average number of posts per user varied significantly (*P* < .001), with more positive users posting 1.9 times and more negative users posting up to four times.

## Discussion

Sentiment regarding CI remains more nuanced than what can be gleaned from this analysis of r/Cochlearimplants, including cultural and ethical issues with these devices; however, this study demonstrates that the sentiment of entries on the r/Cochlearimplants subreddit is generally neutral or positive. Despite this, the sentiment on the site became relatively more negative over time, from 2019 to 2024. The reason for this phenomenon is unknown, but it could suggest that negative feelings towards CIs are increasing as device usage increases. Awareness of sentiment towards CI online can allow healthcare providers to better understand patient perspectives and possibly dispel misinformation spread online. This work requires further exploration to better address the nuances involved in CI care.

The majority of entries were classified as positive or neutral, using VADER and TextBlob, respectively. This suggests that feelings regarding CI on r/Cochlearimplants are largely favorable. The differences between VADER and TextBlob findings may be linked to TextBlob's inability to account for sentiment effects of capital letters, emojis, or repeated words, as discussed in the methods.^22^ Given the anonymity that Reddit provides, sentiments shared on the site may be more authentic than more traditional healthcare assessments.^23^, ^24^ While data gathered was quantitative in nature, content review of the entries revealed that many of the highest voted posts discussed user experience with the CI, photos showing their CI, or discussions regarding pre/postsurgery. This may suggest that r/Cochlearimplants functions as a virtual community of practice, similar to other medical subreddit communities, where users can share information, resources, and social support.^25^, ^26^, ^27^, ^28^

Despite these findings, there was a significant rise in negative sentiment on the subreddit in 2024. The reasons for this proportional increase in negative sentiment on the subreddit could be due to many reasons. First, there is an overall increase in individuals receiving CI.^4^ With this increase in usage, there may be a corresponding increase in negative sentiment. While this analysis was solely quantitative, we are unable to assess the reasons for negative sentiment in a qualitative fashion at this time. However, posts containing negative sentiment regarding CI could be discussing a multitude of parts of the CI process, such as finding a provider, insurance difficulties, preoperative or postoperative complications, aural rehabilitation, device difficulties, and any potentially negative experience. Another possible reason for an increase in proportionally negative sentiment over time may be an increase in overall negative content online related to engagement. Though there is limited data on the overall sentiment of content online, research shows that negative content is often associated with higher engagement, which may motivate users to post further negative content to increase engagement further.^29^, ^30^, ^31^, ^32^ Importantly, entries with negative sentiment in this study did not receive more engagement, possibly suggesting a different mechanism for this trend in sentiment, which requires further evaluation.

Indeed, entries with negative versus positive sentiment had differing characteristics, with negative entries longer on average, but positive entries displaying more engagement. Research has shown that negative sentiment may require further words to expound upon in online reviews.^33^ Meanwhile, higher engagement with positively classified posts (operationalized as upvotes) suggests that supportive or optimistic content may resonate more strongly with CI subreddit participants. Importantly, this is discordant with the general finding discussed above regarding negative content often receiving more engagement. Further qualitative analysis is warranted to explore specific concerns driving negative sentiment, as well as the potential influence of evolving technology, expanded candidacy criteria, and shifting social perceptions of CI.

This study is not without limitations. Given the anonymity of Reddit's user base, we are unable to verify if posters discussing CI history have undergone CI surgery. Furthermore, given that this analysis was performed only on Reddit, the sentiment findings cannot be generalized to other social media platforms, which may be comprised of differing user bases. Furthermore, while the analysis assessed for entries by level of engagement (upvotes), we are unable to account for the algorithm within Reddit which may promote certain posts over others, thus impacting our findings. Given the source of the study as an online site, the analysis excludes individuals less informed about technology and social media platforms as they do not engage on such sites and cannot have their sentiment collected. Finally, this quantitative sentiment analysis is limited and unable to adequately address the ethical, cultural, and political discussions regarding CI.

This analysis found generally neutral and positive sentiment regarding CI on the subreddit r/Cochlearimplants; however, with increasingly more negative sentiment seen on the site over time. Future studies must further examine the reasons for this increase in negative sentiment. Furthermore, more work is required to understand how patients use online platforms such as Reddit for social connection and medical education regarding CI.

## Author Contributions

**Rose Dimitroyannis**, **Terence E. Imbery**, conception and design; **Rose Dimitroyannis**, **Ethan Oliver**, **Ringo Leung**, data acquisition; **Rose Dimitroyannis**, **Ethan Oliver**, **Ringo Leung**, **Rachel Nordgren**, **Terence E. Imbery**, analysis and interpretation; **Rose Dimitroyannis**, **Ethan Oliver**, **Ringo Leung**, **Rachel Nordgren**, **Terence E. Imbery**, drafting the manuscript; **Rose Dimitroyannis**, **Ethan Oliver**, **Ringo Leung**, **Rachel Nordgren**, **Terence E. Imbery**, critical revision; **Terence E. Imbery**, guarantor.

## Disclosures

### Competing interests

None.

### Funding source

None.

## Data Availability

Data collected from this study may be made available upon request from the corresponding author. Reddit posts collected are publicly available through the social media platform.

## References

[1] Deep N , Dowling E , Jethanamest D , Carlson M . Cochlear implantation: an overview. J Neurolog Surg Part B Skull Base. 2019;80(2):169‐177. 10.1055/s-0038-1669411 PMC6438790 30931225

[2] Zeng FG . Celebrating the one millionth cochlear implant. JASA Express Lett. 2022;2(7):077201. 10.1121/10.0012825 36154048

[3] Lin FR . Hearing loss prevalence in the United States. Arch Intern Med. 2011;171(20):1851‐1852. 10.1001/archinternmed.2011.506 22083573 PMC3564588

[4] Hoffman HJ , Dobie RA , Losonczy KG , Themann CL , Flamme GA . Declining prevalence of hearing loss in US adults aged 20 to 69 Years. JAMA Otolaryngol Head Neck Surg. 2017;143(3):274‐285. 10.1001/jamaoto.2016.3527 27978564 PMC5576493

[5] Sorkin DL . Cochlear implantation in the world's largest medical device market: utilization and awareness of cochlear implants in the United States. Cochlear Implants Int. 2013;14(sup1):S12‐S14. 10.1179/1467010013Z.00000000076 PMC3663290 23453146

[6] Zeng FG . Trends in cochlear implants. Trends Amplif. 2004;8(1):1‐34. 10.1177/108471380400800102 15247993 PMC4111484

[7] McRackan TR , Hand BN , Cochlear Implant Quality of Life Development Consortium , Velozo CA , Dubno JR , Dubno JR . Development and Implementation of the Cochlear Implant Quality of Life (CIQOL) functional staging system. Laryngoscope. 2022;132(Suppl 12):S1‐S13. 10.1002/lary.30381 PMC9650765 36082873

[8] Zhan KY , Mazul A , Kallogjeri DL , Buchman CA . Use of diagnostic audiology and cochlear implantation in the US. JAMA Otolaryngol Head Neck Surg. 2024;150(4):353‐354. 10.1001/jamaoto.2023.4738 38386348 PMC10884948

[9] Zhang L , Ding AS , Xie DX , Creighton FX . Understanding public perceptions regarding cochlear implant surgery in adults. Otol Neurotol. 2022;43(3):e331‐e336. 10.1097/MAO.0000000000003439 35147605 PMC10368452

[10] Gao RW , Smith JD , Malloy KM . Head and neck cancer and social media: the patient experience and cancer survivorship. Laryngoscope. 2021;131(4):E1214‐E1219. 10.1002/lary.29074 32886368

[11] Feier JS , Nguyen K , Choi JS . Twitter perspectives on cochlear implantation: sentiment and thematic analysis. Otolaryngol Head Neck Surg. 2023;169(3):642‐650. 10.1002/ohn.292 36939425

[12] Proferes N , Jones N , Gilbert S , Fiesler C , Zimmer M . Studying reddit: a systematic overview of disciplines, approaches, methods, and ethics. Social Media + Society. 2021;7(2). 10.1177/20563051211019004

[13] Suler J . 2004. The online disinhibition effect. Cyberpsychol Behav 7. 2004;3:321‐326. 10.1089/1094931041291295 15257832

[14] Ma X , Hancock J , Naaman M . Anonymity, intimacy and self‐disclosure in social media. In Proceedings of the 2016 CHI Conference on Human Factors in Computing Systems (CHI ‘16). Association for Computing Machinery; 2016:3857‐3869. 10.1145/2858036.2858414

[15] Microsoft Corporation . Microsoft Excel; 2018. https://office.microsoft.com/excel

[16] R Core Team . *R: A language and environment for statistical computing*. Published online 2022. https://www.R-project.org/

[17] Heinzen E , Sinnwell J , Atkinson E , Gunderson T , Dougherty G . arsenal: An Arsenal of ‘R’ Functions for Large‐Scale Statistical Summaries. R package version 3.6.3; 2021.

[18] Wickham H . ggplot2: Elegant Graphics for Data Analysis. Springer‐Verlag; 2016.

[19] “G. van Rossum, Python tutorial, Technical Report CS‐R9526, Centrum voor Wiskunde en Informatica (CWI), Amsterdam, May 1995.”

[20] Loria S . Textblob Documentation. Release 0.15, 2; 2018.

[21] Roehrick K . vader: Valence Aware Dictionary and sEntiment Reasoner (VADER). R package version 0.2.1; 2020. https://CRAN.R-project.org/package=vader

[22] Patil H . “Textblob vs Vader for Sentiment Analysis.” Medium, Medium, 2 Apr. 2023, medium.com/@hhpatil001/textblob-vs-vader-for-sentiment-analysis-9d36b0b79ae6

[23] Agarwal N , Rahman A , Jacobs R , et al. Patient perception of scoliosis correction surgery on Instagram. Neurosurg Focus. 2021;51(5):E6. 10.3171/2021.8.FOCUS201015 34724639

[24] Xie DX , Boss EF , Stewart CM . An exploration of otolaryngology in the Reddit community. Laryngoscope. 2022;132:284‐286. 10.1002/lary.29696 34319587

[25] Swift L . Online communities of practice and their role in educational development: a systematic appraisal. Community Pract. 2014;87(4):28‐31 24791455

[26] Li LC , Grimshaw JM , Nielsen C , Judd M , Coyte PC , Graham ID . Evolution of Wenger's concept of community of practice. Implement Sci. 2009;4:11. 19250556 10.1186/1748-5908-4-11 PMC2654669

[27] Dimitroyannis R , Thodupunoori S , Leung R , et al. Sentiment analysis of transsphenoidal surgery in the Cushing's Subreddit. J Neurol Surge Part B: Skull Base. 2024;86(4):488‐494. 10.1055/a-2360-9748 PMC12227243 40620631

[28] Dimitroyannis R , Fenton D , Thodupunoori S , et al. “Just got diagnosed; what worked for you?”: a mixed‐methods analysis of treatment experiences in the prolactinoma Subreddit. J Neurol Surg Part B: Skull Base. 2024;86(4):480‐487. 10.1055/a-2358-9228 PMC12227248 40620626

[29] Robertson CE , Pröllochs N , Schwarzenegger K , Pärnamets P , Van Bavel JJ , Feuerriegel S . Negativity drives online news consumption. Nat Hum Behav. 2023;7(5):812‐822. 10.1038/s41562-023-01538-4 36928780 PMC10202797

[30] Watson J , van der Linden S , Watson M , Stillwell D . Negative online news articles are shared more to social media. Sci Rep. 2024;14(1):21592. 10.1038/s41598-024-71263-z 39285221 PMC11405697

[31] Schöne JP , Garcia D , Parkinson B , Goldenberg A . Negative expressions are shared more on Twitter for public figures than for ordinary users. PNAS Nexus. 2023;2(7):pgad219. 10.1093/pnasnexus/pgad219 37457891 PMC10338895

[32] Stella M , Ferrara E , De Domenico M . Bots increase exposure to negative and inflammatory content in online social systems. Proc Natl Acad Sci. 2018;115(49):12435‐12440. 10.1073/pnas.1803470115 30459270 PMC6298098

[33] Takumi K. “Negative Sentiments Make Review Sentences Longer: Evidence from Japanese Hotel Review Sites: Integrated Uncertainty in Knowledge Modelling and Decision Making.” Guide Proceedings, 2 Nov. 2023, 10.1007/978-3-031-46781-3_24

